# CHALLENGES IN PRE-REGISTRATION AND APPLYING OPEN SCIENCE PRINCIPLES WHEN USING SECONDARY OR LONGITUDINAL DATA

**DOI:** 10.1093/geroni/igz038.1482

**Published:** 2019-11-08

**Authors:** Daniel K Mroczek

**Affiliations:** Northwestern University, Chicago, Illinois, United States

## Abstract

The application of open science, preregistration, and transparency principle is challenging when using existing data, including ongoing long-term longitudinal data. The goal is to distinguish clearly between exploratory and confirmatory research, but in the context of archival or longitudinal work there are risks associated with prior knowledge that has been obtained from these secondary sources. That said, new principles are being developed, including specialized pre-registration templates, that can guide the application of open science and transparency ideas to longitudinal and other secondary data, thereby increasing credibility of such work. These include: 1) disclosure of prior knowledge about a given dataset, ranging from “never worked with these data” to having multiple publications, in the pre-registration, 2) use of hold-out subsamples that can be used for validation or confirmatory purposes, and 3) making more clear what research questions are exploratory and confirmatory.

